# Implications of practical activities in the Skills and Simulation Laboratory on students’ motivation and feelings

**DOI:** 10.1590/1518-8345.6397.3903

**Published:** 2023-05-15

**Authors:** Barbara Casarin Henrique-Sanches, Luis Sabage, Raphael Raniere de Oliveira Costa, Rodrigo Guimarães dos Santos Almeida, Roberson Antequera Moron, Alessandra Mazzo

**Affiliations:** 1 Universidade de São Paulo, Escola de Enfermagem de Ribeirão Preto, Centro Colaborador de la OPS/OMS para el Desarrollo de la Investigación en Enfermería, Ribeirão Preto, SP, Brasil.; 2 Becaria de la Coordenação de Aperfeiçoamento de Pessoal de Nível Superior (CAPES), Brasil.; 3 Universidade de São Paulo, Faculdade de Odontologia de Bauru, Bauru, São Paulo, Brasil.; 4 Universidade Federal do Rio Grande do Norte, Escola Multicampi de Ciências Médicas do Rio Grande do Norte, Caicó, Rio Grande do Norte, Brasil.; 5 Universidade Federal de Mato Grosso do Sul, Instituto Integrado de Saúde, Campo Grande, Mato Grosso do Sul, Brasil.

**Keywords:** Learning, COVID-19, Education, Medical, Students, Medical, Simulation Training, Motivation, Aprendizaje, COVID-19, Educación Médica, Estudiantes de Medicina, Entrenamiento por Simulación, Motivación, Aprendizagem, COVID-19, Educação Médica, Estudantes de Medicina, Treinamento por Simulação, Motivação

## Abstract

**Objective:**

to verify the implications of practical activities in the Skills and Simulation Laboratory on the motivation and feelings expressed by undergraduate students when returning to face-to-face activities after the social isolation caused by COVID-19 pandemic.

**Method:**

a quasi-experimental study, with a single group and of the pre- and post-test type, carried out through an educational intervention based on skills training on medication administration and venipuncture, with medical students from a Brazilian public university. The sample was comprised by 47 students. The instruments of students’ characterization and self-perceived feelings and the Situational Motivation Scale were used for data collection.

**Results:**

in the sample, 98% mentioned the lack of practical activities during the pandemic. The most frequently described feeling was anxiety. After carrying out the activity, there was a change in the frequency of expressed feelings, although there was no significant change in motivational levels. External Regulation (5.1 - 5.6), Identified Regulation (6.1 - 6.4) and Intrinsic Motivation (5.6 - 6.0) presented high results, showing similarity to the feelings reported by the learners.

**Conclusion:**

motivation is essential for effective learning and the use of active methodologies reinforces skills built in an affective way in the students facing the learning process.

## Abstract (es)

**Objetivo:**

verificar las implicaciones de las actividades prácticas en el Laboratorio de Habilidad y Simulación relacionadas con la motivación y los sentimientos expresados por los estudiantes universitarios cuando regresan a las actividades presenciales luego del aislamiento social ocasionado por la pandemia del COVID-19.

**Método:**

estudio cuasiexperimental, con un solo grupo y del tipo pre y postest, realizado mediante una intervención educativa basada en el entrenamiento de habilidades en administración de medicamentos y venopunción, con estudiantes de medicina de una universidad pública brasileña. La muestra estuvo conformada por 47 estudiantes. Para la recolección de datos se utilizaron los instrumentos de caracterización y autopercepción de los individuos y la Escala de Motivación Situacional.

**Resultados:**

en la muestra, el 98% mencionó la falta de actividades prácticas durante la pandemia. El sentimiento descrito con mayor frecuencia fue la ansiedad. Después de realizar la actividad, hubo un cambio en la frecuencia de los sentimientos expresados, aunque no hubo un cambio significativo en los niveles de motivación. Regulación Externa (5,1 – 5,6), Regulación Identificada (6,1 – 6,4) y Motivación Intrínseca (5,6 – 6,0) presentaron resultados altos, demostrando similitud con los sentimientos relatados por los estudiantes.

**Conclusión:**

la motivación es fundamental para un aprendizaje efectivo y el uso de metodologías activas refuerza de manera efectiva las habilidades construidas en los estudiantes frente al proceso de aprendizaje.

## Abstract (pt)

**Objetivo:**

verificar as implicações das atividades práticas no Laboratório de Habilidade e Simulação relacionado à motivação e os sentimentos expressos pelos estudantes universitários quando regressam às atividades presenciais após o isolamento social causado pela pandemia da COVID-19.

**Método:**

estudo quase-experimental, com um único grupo, do tipo pré e pós-teste, realizado por meio de uma intervenção educacional baseada no treino de habilidades de administração de medicamentos e punção venosa, com estudantes de medicina de uma universidade pública brasileira. A amostra foi composta por 47 estudantes. Para a coleta de dados, foram utilizados os instrumentos de caracterização dos sujeitos e sentimentos autopercebidos pelos estudantes e a Escala de Motivação Situacional.

**Resultados:**

na amostra, 98% referiram falta de atividades práticas durante a pandemia. O sentimento mais frequentemente descrito foi a ansiedade. Após a realização da atividade, observou-se uma mudança na frequência dos sentimentos expressos, embora não se tenha verificado uma mudança significativa nos níveis motivacionais. A Regulação Externa (5,1 – 5,6), a Regulação (6,1 – 6,4) e a Motivação Intrínseca (5,6 – 6,0) apresentaram resultados elevados, demostrando semelhança com os sentimentos relatados pelos alunos.

**Conclusão:**

a motivação é essencial para uma aprendizagem eficaz e a utilização de metodologias ativas reforça as habilidades construídas de uma forma efetiva nos estudantes frente ao processo de aprendizagem.

**Highlights:**

**(1)** Desires and needs for face-to-face LHS activities during and after isolation.

**(2)** Feeling of fear related to the risk of contamination during activities.

**(3)** Self-determined motivational profiles, linked to identified and intrinsic motivation.

**(4)** Motivation to practice activities is related to accomplishment, pleasure, and satisfaction.

## Introduction

The pandemic caused by Coronavirus Disease 2019 (COVID-19) affected millions of people around the world, causing deaths that impacted on various sectors of social life ^( 1)^ . In education, it caused disruption in the teaching-learning process, which, as a matter of emergency, was implemented remotely and in different environments. The students experienced social isolation in an impactful way, largely impairing the health area courses and causing harms in knowledge, skills and attitudes, as well as in their satisfaction and motivation with the adaptations imposed on the educational process ^( 2)^ . 

Among the various methods used in teaching and learning with health professionals, the use of the Skills and Simulation Laboratory (SSL) has been highlighted due to the development of varied skills and capabilities ^( 3)^ . 

Clinical simulations can take place in a variety of physical environments, but it usually focuses its activities on the SSL. In these environments, the activities are carried out with the greatest realism ^( 4)^ possible, mimicking the real clinical practice, which invokes feelings, knowledge, skills and attitudes in the learner that can be worked on and exhaustively repeated so that they feel increasingly confident in their actual clinical practices ^( 5)^ . 

The simulation activities in the SSL are associated with the students’ personal involvement, which can be impacted by their state of motivation ^( 6)^ . 

Motivation can occur through small changes at the situational level, resulting from a successive number of interactions with the environment and repeatedly internalized ^( 7)^ , which gives meaning to knowledge and to previous experiences. 

In this study, the Self Determination Theory (SDT) was employed as theoretical reference. SDT points out that socio-contextual conditions contribute to or inhibit natural processes of self-motivation and healthy psychological development. Therefore, factors related to methodology and teaching method trigger processes that can increase or decrease intrinsic motivation, self-regulation, and well-being ^( 8)^ . 

The formal nucleus of SDT is currently composed of six sub-theories, among which we highlight the Cognitive Evaluation Theory and the Organismic Integration Theory ^( 8)^ . 

Cognitive Evaluation Theory is structured upon intrinsic motivation and extrinsic motivation. Intrinsic motivation is one in which the activity itself generates satisfaction, without external interests or related rewards. Extrinsic motivation refers to performing an activity to achieve an external result, with known rewards and recognition ^( 8)^ . 

Organismic Integration Theory addresses a set of broad contextual factors related to the internalization and regulation of extrinsic motivation behaviors. Internalization is distinguished into regulatory modalities; namely, Non-Regulation, External Regulation, Introjected Regulation, Identified Regulation, Integrated Regulation, and Intrinsic Regulation ^( 8)^ . In this study, External Regulation and Identified Regulation were highlighted. External Regulation relates to less autonomous and extrinsically motivated behaviors based on satisfaction of external demands or contingent rewards. In Identified Regulation, although the objective is external, it becomes accepted by the individual as important, generating conscious valorization of a behavior. Demotivation is the result of the lack of internalization of a specific external regulator to perform a behavior, which leads to disorganized, impulsive, or passive responses ^( 8)^ . 

During the pandemics, regardless of the engagement, increased activities by the teachers, and facilitators were offered only in in the virtual environment, face-to-face practices in the SSL could not be experienced.

In this context, this study had the objective to verify the implications of the SSL in the students’ motivation on the return to face-to-face activities after the period of social isolation caused by the COVID-19 pandemic.

## Method

### Study design

A quasi-experimental study, with a single group and of pre-test and post-test type ^( 9)^ , carried out in a second-year medical student class. 

### Scenario

This study was conducted at a public university in Bauru-SP, Brazil, from July 26 to 30, 2021, in a medical course, which has its political pedagogical project based on active methodologies, with several active teaching methods and a structured laboratory of skills and simulation.

SSL is a space that has a physical area with different environments and a set of simulators, which are diverse in complexity and fidelity for the development of various clinical skills and real-life scenarios. In some activities, it also counts on the use of moulage and the participation of actors ^( 10)^ . 

On the return of the in person didactic activities of the course, which were interrupted due to the need for isolation due to the COVID-19 pandemic, the activities in the SSL were the first ones to restart, strictly following the biosafety rules for the protection of the students ^( 10)^ . 

### Population and sample

The students that participated on the study had never personally frequented the SSL.

Students from the 2 ^nd^ year of the course, regularly enrolled, older than 18 years, who started graduation in 2020, had had previous contact with the course activities exclusively remotely (due to the pandemic), and who participated in all the activities proposed in this study were included in the sample. Among the 53 students participating in the activity, 47 completed all the study stages—they were present in the practices at the Skills and Simulation Laboratory and answered the data collection instruments before and after the practice; therefore, they were included in the sample. 

### Study variables

The variables compared consisted of the students’ self-perceived feelings about returning to face-to-face activities, self-perceived feelings about the risk of contamination by SARS-CoV-2 while carrying out the activity at the SSL, and the students’ motivational profile for learning, all before and after carrying out the proposed SSL activity.

### Data collection instruments

A) Instrument for the characterization of students and self-perceived feelings, consisting of a questionnaire with open and closed questions, related to age, gender, course period, as well as experiences and feelings self-reported by students, considering both the period of social isolation, regarding the resumption of face-to-face activities in relation to the risk of contamination by COVID-19 during activities at SSL.

B) Situational Motivation Scale ^( 11)^ (Cronbach’s Alpha ≥ 0.7). The instrument is intended to assess situational motivation in an educational context. It is a 16-item instrument divided into 4 categories: Intrinsic Motivation (“Because I think this activity is interesting”), Identified Regulation (“Because it’s for my own good”), External Regulation (“Because I can do it”) and Demotivation (“There might as well be good reasons for doing this activity, but I don’t see any”). The answers to the items are given on a Likert-type scale ranging from 1 (Does not match at all) to 7 (Exact match). 

### Study development

Upon returning to face-to-face activities, the 2 ^nd^ year students performed, as their first activity at SSL, the administration of parenteral medication. This theme was chosen by the professors because, following the activity, the students started to participate in the vaccination campaign against COVID-19. 

In the SSL, before the activities began, the objectives were explained, and the students were invited to participate in the study. Those who accepted, formalized their acceptance by signing the Informed Consent Form (ICF). After completing the ICF, students were assigned a participant number and each responded to the instrument for characterizing students and self-perceived feelings and the Situational Motivation Scale ^( 11)^ . Afterwards, the students participated in skills training and scenario development on how to administer parenteral medications safely in groups of no more than 10 students. 

The activities were developed based on the literature review; and were planned, built, and validated regarding structure and content for experts, in addition to being tested before their practical application. They were developed by calibrated facilitators, nurses, and medical course professors; were preceded by a study in a virtual learning and discussion environment and, after completion, feedback and debriefing sessions were carried out. In the activities, low and medium fidelity simulators were used, with realism - when puncturing a vein, there was blood return or, when puncturing the dermis, the student felt the sensation of tissue transfixation, among many others. During the activities, the students were supported by scripts prepared by the professors.

The activities were promoted for 5 days. On the last day, the students answered again the questions related to self-perceived feelings from the instrument and the Situational Motivation Scale ^( 11)^ . 

### Data processing, analysis and disclosure

The data from the instruments for the participants’ characterization and the Situational Motivation Scale ^( 11)^ were encrypted, transferred to a Microsoft Excel ^®^ spreadsheet, and analyzed in IBM SPSS ^®^ statistical software version 24 (IBM, Inc, Chicago, IL), with descriptive analyzes performed by absolute (n) and relative (%) frequencies. The Situational Motivation Scale ^( 11)^ was analyzed in four categories, as proposed by the author. The Student’s t test was used for sample comparison. Pearson’s correlation test was also performed between the scale factors. 

### Ethical procedures

This study has ethical authorization under opinion No. 4,843,772 of the Research Ethics Committee of *Faculdade de Odontologia da Universidade de São Paulo* - FOB-USP. The participants expressed their acceptance of the research by signing the ICF. There were no refusals to participate in the study. 

## Results

Among the 47 participants, 24 (51.1%) were female and 23 (48.9%) were male, aged between 18 and 29 years old, with a mean of 21 years.

When asked about the need for practical activities during the quarantine period, 46 (98.0%) students reported that they felt this need and 45 (95.7%) performed one or more practical activities at their homes during the period (measurement of vital signs and physical examination practices in friends or in the family itself).

When resuming the SSL face-to-face activities after the period of social isolation and immediately at the end of the initial activities performed, the students were asked to express their feelings in one word. Self-perceived feelings are shown in Table 1. 

**Table 1 -:** Self-perceived feelings of the students after the period of social isolation before and after the initial SSL ^*^ activities. Bauru, SP, Brazil, 2021

Before feelings	Fr ^†^	% ^‡^	After feelings	Fr ^†^	% ^‡^
Anxiety	12	25.5	Happiness	23	49.0
Happiness	12	25.5	Excitement	5	10.7
Excitement	3	6.4	Anxiety	5	10.7
Hope	3	6.4	Enthusiasm	3	6.4
Expectation	3	6.4	Satisfaction	2	4.3
Relief	2	4.3	Fondness	1	2.1
Enthusiasm	2	4.3	Good	1	2.1
Gratitude	2	4.3	Curiosity	1	2.1
Fear	2	4.3	Fun	1	2.1
Good	1	2.1	Hope	1	2.1
Lack of preparation	1	2.1	Gratitude	1	2.1
Euphoria	1	2.1	Positive	1	2.1
Motivation	1	2.1	Fulfillment	1	2.1
Positivity	1	2.1	Willingness	1	2.1
Confidence	1	2.1			
**Total**	**47**	**100.0**	**Total**	**47**	**100.0**

^*^SSL = Skills and Simulation Laboratory; ^†^Fr = Frequency; ^‡^% = Percentage

After the initial surveys, the students experienced the intensive practices of the period and, before and after the activities, they answered which feelings were related to the risk of contamination by SARS-CoV-2 during the SSL practice and in the Situational Motivation Scale ^( 11)^ . The self-perceived feelings related to resuming the face-to-face activities and the risk of contamination by SARS-CoV-2 before and after the activities are described in Table 2. 

**Table 2 -:** Self-perceived feelings expressed regarding the risk of contamination by SARS-CoV-2 ^*^ before and after performing the SSL ^†^ practical activities. Bauru, SP, Brazil, 2021

Before feelings	Fr ^‡^	% ^§^	After feelings	Fr ^‡^	% ^§^
Confidence	13	27.7	Fear	15	31.9
Fear	12	25.5	Peace of mind	14	29.8
Insecurity	4	8.5	Confidence	7	14.9
Concern	4	8.5	Controlled	3	6.4
Peace of mind	4	8.5	Apprehension	2	4.3
Worry	3	6.4	Insecurity	2	4.3
Controlled	2	4.3	Anguish	1	2.1
Moderate risk	2	4.3	Concern	1	2.1
Apprehension	1	2.1	Worry	1	2.1
Effort required	1	2.1	Necessary risk	1	2.1
No feelings	1	2.1			
**Total**	**47**	**100.0**	**Total**	**47**	**100.0**

^*^SARS-CoV-2 = Severe Acute Respiratory Syndrome Coronavirus 2; ^†^SSL = Skills and Simulation Laboratory; ^‡^Fr = Frequency; ^§^% = Percentage

The Situational Motivation Scale ^( 11)^ showed good reliability (α=0.714) in the studied sample. The highest values measured by the instrument were in the Identified Regulation domain, both before and after the initial activities, and the lowest were found in the Demotivation domain, also in both periods. Table 3 shows the results of the instrument in relation to the students’ motivation, measured before and after carrying out the practical activities in the SSL after a prolonged period of social isolation. 

**Table 3 -:** Motivational profile for the students’ learning ^( 11)^ before and after the first SSL ^*^ activities after isolation. Bauru, SP, Brazil, 2021

Period	Domains	Min ^†^	Max ^‡^	Mean	Standard Deviation
Before	Identified Regulation	4.0	7.0	6.1	0.747
	Intrinsic Motivation	3.7	7.0	5.6	0.769
	External Regulation	2.0	7.0	5.1	1.349
	Demotivation	1.0	3.50	1.4	0.659
After	Identified Regulation	4.6	7.0	6.4	0.659
	Intrinsic Motivation	3.7	7.0	6.0	0.710
	External Regulation	1.3	7.0	5.6	1.526
	Demotivation	1.0	4.0	1.2	0.623

^*^SSL = Skills and Simulation Laboratory; ^†^Min = Minimum; ^‡^Max = Maximum

The sample presented normal distribution (Kolmogorov-Smirnov test > 0.05); therefore, to compare the students’ motivation before and after the activity, the *Student*’s t test was used. The results show that there were no significant differences between the results presented in the students’ motivation before and after the practice performed at resumption of the activities. 

To assess the correlation between the Situational Motivation Scale ^( 11)^ values, at the moments before and after the practice performed at resumption of the activities, Pearson’s Correlation Coefficient was used. In this regard, the results showed a strong correlation (0.7 - 0.9) in the following domains Identified Regulation before and after, Intrinsic Motivation before and after, and External Regulation before and after, and a weak correlation (0.3 - 0.5) in the Demotivation domain before and after the practice performed at resumption of the activities, as shown in Table 4. 

**Table 4 -:** Correlation between the general domains of the Situational Motivation Scale ^( 11)^ at resumption of the face-to-face activities before and after performing the SSL ^*^ in-person activities. Bauru, SP, Brazil, 2021

Correlated Domains	ρ ^†^
Identified Regulation before *vs*. ^‡^ Identified Regulation after	0.832
Intrinsic Motivation before *vs*. ^‡^ Intrinsic Motivation after	0.787
External Regulation before *vs*. ^‡^ External Regulation after	0.709
Demotivation before *vs*. ^‡^ Demotivation after	0.321

^*^SSL = Skills and Simulation Laboratory; ^†^ρ = Pearson’s Correlation Coefficient; ^‡^ *vs*. = *Versus*

## Discussion

Over the last few years, traditional education has increasingly been replaced in the training of health professionals by methodologies and methods that provoke and immerse the students in their learning process, valuing the context in which they are inserted, developing greater self-confidence and turning them into more competent professionals. In this context, several teaching environments needed to be revised, remodeled and, in some places, instituted. Among them, the SSL has stood out for the amount of processes it can add and the variety of simulated situations it can provide.

The participants of this study, which sought to investigate the importance of the SSL in the motivation of medical students resuming face-to-face activities, were students of both genders, young adults, who reported difficulties in apprehension and in the training of their course practices during the interruption of face-to-face activities. These factors may be related to changes in the methods and methodologies used in the period, in addition to the stress, uncertainty, and psychic distress associated with dealing with the COVID-19 pandemic, already reported by other medical students ^( 12- 13)^ . 

Among the feelings mentioned in the resumption of face-to-face activities, positive issues such as happiness, excitement, expectations, hope, gratitude and enthusiasm, relief and positivity stood out, as well as negative feelings of fear. The feelings have been discussed in the COVID-19 pandemic context in various teaching sciences and modalities. The positive ones, possibly, caused by resumption of the routine, coexistence and institutional support, which, when interrupted during the period of social isolation due to COVID-19, caused, as in this sample, illness, stress, anxiety and depression in several students ^( 14)^ . 

As shown in Table 1, the students’ feelings before the activities started were filled with anxiety and happiness, and after the activities, happiness. Possibly, happiness is related to the return to the face-to-face activities, to the meeting with the group, the first in person contact with the course, its professors and colleagues, previously only known virtually; antagonistically, considering anxiety about contact with other people and even with unfamiliar surroundings in such a challenging time faced during the pandemic ^( 15)^ . 

Among the medical students, some authors highlight that the anxiety levels during the pandemic were high (nearly 28% of the students) which may have been caused by issues related to concerns about academic progress, distance learning, the intensity of activities, among others. However, the same authors reported that, although elevated during the COVID-19 pandemic, medical students’ anxiety was lower than in a non-pandemic period. Generally, the anxiety of medical students is higher than that of other students in the health field, which is related to the fact that these students have an intensified study routine. During the pandemic, the study routine was replaced by the online routine, which may have benefited this process ^( 16)^ . 

Regarding the risk of contamination by COVID-19, as shown in Table 2, there was an inversion in students’ self-perceived feelings in the sample before and after initiation of the activities, regarding confidence and fear. Table 2 emphatically points out that, after the activities developed in this study, sample’s self-perceived feeling was tranquility. Confidence and peace of mind are probably related to the strict biosafety practices that have been incorporated into the SSL, locus where the activities were carried out ^( 10)^ . Fear is a defense feeling that leads individuals to a state of alert, however, when too much present, it can impair their daily activities ^( 17)^ . 

In this sample, fear was more evident after the work practices, which can be explained by the experience of biosafety issues and proximity to medical and nursing professors working in the COVID-19 clinical fields, and to other professionals and colleagues who reported stories of losses. The most recent studies have indicated that, among the students, fear during the teaching activities in the COVID-19 pandemic has been continuous due to the contamination risk, although it has apparently been minimized over time ^( 18- 19)^ . 

Regarding motivation, it is known that the environment exerts a direct influence on the students’ motivation for learning and that, when we compare face-to-face teaching with distance learning, in the in-person modality, the students tend to be more participative and involved in the activities, which results in greater motivation ^( 20)^ . In this context, it should be noted that the SSL is an environment that includes human, physical and material resources that add light and hard technology, with broad intensity, consistent with the students’ curriculum. In this environment, the learning objectives direct the use of resources under the facilitators’ creativity based on a confidentiality and respect contract, teaching is playful. When fully associated, creativity and human and material resources trigger a high level of realism, which means that the frequent or uncommon activities of the clinical practice can be trained to exhaustion. 

As detected by the Motivational Scale ^( 11)^ Table 3, it was possible to observe in the sample that the lowest values attributed to the instrument by the students, before and after resumption of the activities, both in the maximum and minimum scores, were in the Demotivation domain, and that the highest values attributed presented a decreasing order in the Identified Regulation, Intrinsic Motivation, and External Regulation items. In the same results, it is also highlighted that External Regulation was the domain that presented the greatest variation across the values assigned by the students, in both periods: before the activity and after the activity ^( 8)^ . 

The students may lack motivation or be motivated based on external or internal factors. Identified Regulation constitutes a positive evaluation of the sample in relation to the activities conducted in the SSL during the study. Intrinsic Motivation reveals that there was an innate interest in it, and the two factors together constitute the Autonomous Motivation ^( 8)^ . Autonomous Motivation is considered the most desirable type of motivation in the students, as it can be related to better learning results, high performance, commitment, and well-being ^( 21- 22)^ . Some authors argue that medical students with higher levels of Autonomous Motivation are more likely to offer more independent care to the patients, which contributes benefits in general ^( 23)^ . 

External Regulation is related to the external pressures, to the *status* provided by an activity ^( 22)^ and, in this sample, it was this domain that presented the greatest change between the minimum and maximum values among the students, which can be explained by the return to group activities, by the feelings of fear expressed due to the COVID-19 pandemic, or by the anxiety shown, among many others Table 2 that deserve to be further investigated in SSL environments. On the other hand, the positive feelings related to the resumption of the in-person activities exerted a direct influence on the emotional systems in the processes of associative learning and memory through emotional networks, resulting in knowledge retention and cognitive gain, facilitating learning, and mediating self-motivation and satisfaction ^( 24)^ . 

When investigating the preference for face-to-face return to carry out clinical activities during the COVID-19 pandemic in medical students, one study showed that students who opted for face-to-face resumption had higher levels of Intrinsic Motivation, indicating a greater degree of self-determined regulatory style of motivation for learning when compared to students who preferred to remain away from the clinical environment ^( 25)^ . 

It is important to understand that motivation is a dynamic process, which can be modified both by external and internal factors ^( 22)^ . However, in this sample, there were no significant changes in the domains of the instrument used ( *t* Test ≥ 0.05) when comparing the moments before and after the activity. The same was corroborated by the correlation found between the two periods Table 4. The correlation before and after the activity between Identified Regulation was strong and positive. Between Intrinsic Motivation and External Regulation, it was moderate and positive, which shows that the possibility of performing the activity was motivating, and, after the activity, this attribute was even greater. This fact can be observed in the data available in Table 4. 

### Implications for practice and research

Although the results of this study concern a small number of students, a single set of activities and a moment that can be considered special in the trajectory of each student — the COVID-19 pandemic —, factors that can be indicated as limiting factors for this research, the results show that students’ motivation for learning may be related to face-to-face practices, teaching environments, the feeling of belonging, the simulated teaching method, which should be used by institutions, teachers and facilitators, and explored further in other investigations.

## Conclusion

Motivation is essential for effective learning and to train proactive professionals. In this study, it was possible to observe that, although the motivational levels did not undergo significant changes after carrying out the activities, Intrinsic Motivation, External Regulation and Identified Regulation remained high, evidencing the students’ need and aspirations for the resumption of the face-to-face activities after months without practices or social contact. In view of the findings, the importance of studies analyzing effective approaches and methods that enhance students’ learning in order to adopt the necessary interventions in the teaching-learning process is highlighted.
